# Association between social support and pregnancy stress: a cross-sectional study of neighbors’ interactions

**DOI:** 10.1186/s42506-022-00113-5

**Published:** 2022-09-12

**Authors:** Sadaf Abdi, Mahbobeh Faramarzi, Zinatossadat Bouzari, Mohammad Chehrazi, Maria Esfandyari

**Affiliations:** 1 Student Research Committee, Babol University of Medical Sciences, Babol, Iran; 2 Social Determinants of Health Research Center, Health Research Institute, Babol University of Medical Sciences, Babol, Iran; 3 Cancer Research Center, Health Research Institute, Babol University of Medical Sciences, Babol, Iran; 4 Department of Biostatistics and Epidemiology, School of Public Health, Babol University of Medical Sciences, Babol, Iran

**Keywords:** Social support, Neighbors, Family, Friends, Pregnancy stress

## Abstract

**Background:**

Pregnancy is associated with substantial stressful experiences. There are controversies concerning the positive and negative roles of social support during pregnancy. This research aimed to study the association of social support with the pregnancy-related stress.

**Methods:**

In the current cross-sectional research, 200 pregnant women were recruited through convenience sampling from two teaching hospitals affiliated with Babol University of Medical Sciences and a private obstetric clinic. The women completed two self-reported questionnaires during prenatal care appointments. The questionnaires included the Revised Prenatal Distress Questionnaire (NuPDQ) and Social Support Questionnaire (SSQ).

**Results:**

A significantly positive association was observed between the social support of neighbors and the total score of pregnancy stress (*P*<0.001), as well as the scores of its four subscales, namely medical problems (*P*<0.001), parenting (*P*=0.25), infant health stress (*P*=0.006), and pregnancy symptoms (*P*=0.001). Based on the linear regression models, the social support of neighbors was significantly related to the medical problem-associated stress in pregnant women (*β* = 0.147, 95% CI: 0.14 to 0.62, *p =* 0.047), pregnancy symptom (*β* = 0.203, 95% CI: −0.01 to 0.327, *p =* 0.017), and fear of childbirth (*β* = 0.164, 95% CI: 0.05 to 0.38, *p =* 0.046). Furthermore, the neighbors’ social support (β = 0.172, 95% CI: 0.02 to 0.32, *p =* 0.04) and the total score of social support (*β* = 0.155, 95% CI: 0.01 to 0.304, *p =* 0.046) were significantly associated with the total score of pregnancy stress.

**Conclusions:**

Neighbors’ support, as a component of social support, was found to be significantly related to pregnancy stress. This study recommends that healthcare providers consider the positive and negative impacts of social support during the pregnancy period.

## Background

Social support is a multi-dimensional concept classified into emotional support, network, esteem, material resources, and informational support [1]. Social support may have positive and negative effects on mental health [2]. The literature has mostly focused on the positive aspects of social support [3, 4]. A study in China reported that social support could affect the health outcomes by facilitating stress management [4]. However, evidence has revealed a number of disadvantages related to social support [2].

Pregnancy is associated with substantial physiological and psychological changes as well as stressful experiences [5]. Pregnancy stress involves worries about relationship, parental period, physical changes, delivery, as well as infant health, and his/her future care [6]. A higher level of stress during this period increases the prevalence of mental disorders [7]. Women who suffer from more stress during pregnancy are more prone to experience poor outcomes for both themselves and their infant [8, 9].

There are controversies about the positive and negative roles of social support during pregnancy. Negative social support refers to negative social interactions, including discouraging emotional expression, invading privacy, criticizing, and not offering assured help [10]. Most available data suggest that women with more social support are less likely to experience mental problems in pregnancy [11–13]. Additionally, partner and family support for pregnant women may result in a reduction in mental disorders following delivery [14]. Due to a lack of information on the impact of positive and negative characteristics of social support on pregnancy stress, the present research filled the gap in the literature, delving into the function of social support in such stress. This research aimed to study the association of social support of friends, families, and neighbors with pregnancy stress.

## Methods

### Study design and procedures

We carried out a cross-sectional investigation at two teaching hospitals affiliated with Babol University of Medical Sciences in addition to a private obstetric clinic, from September 2018 to April 2019. The inclusion criteria were pregnant women aged above 18 years and with a minimum of five years of education. Women with a high-risk pregnancy, including hypertension, diabetes, maternal bleeding, and preterm labor, on top of those with a history of serious medical conditions, psychiatric problems, and drug abuse /alcohol addiction were excluded.

In order to recruit the subjects, we used convenience sampling method. Prior to starting the research, we calculated the ratio of pregnancy stress in a pilot study (*P* =0.6). The sample size was thus assessed as 188 with respect to *α*=0.05, *P*=0.6, and *d*=0.07. The researchers increased the number to 200 to compensate for the incomplete and missing data $n=\frac{{\left({Z}_{1-\frac{\alpha }{2}}\right)}^2p\left(1-p\right)}{(d)^2}$.

Two important influential factors that should be considered include socio-economic status and living in cities or villages. In order to control these factors, the sampling location of the pregnant women was taken into consideration so that the effect of these factors on the results could be controlled. Thus, the sampling was performed from two secondary and tertiary perinatal hospital centers (Yahyanejad and Ayatollah Rohani) so that the rural and low-income women could also be sampled. Furthermore, a private practice was randomly selected to include women with a high level of socio-economic status in the sampling. Thus, 100 rural women were selected equally from both hospitals (50 patients per hospital) in addition to 100 urban women from private offices, to control both socio-economic status and place of residence.

A total of 245 pregnant women were asked to participate in the study via convenient sampling. They were recruited by a member of the research team (first author) and two midwives, who interviewed the women and gathered their medical and obstetric histories, as well as demographic information, in order to determine their obstetric risks and appropriateness based on the inclusion criteria. Furthermore, the midwives gave the ladies a brief explanation about the study’s objective and how to fill out the surveys. The midwives entered every eligible women in the study. Eventually, 200 eligible women (100 women living in village and 100 in city) provided written informed consent. Accordingly, the response rate was 81.6%.

All the 200 participants completed two self-reported questionnaires in their prenatal care appointments. These questionnaires included the Social Support Questionnaire (SSQ) and Revised Prenatal Distress Questionnaire (NuPDQ) (15-17).

The Medical Ethics Committee of Babol University of Medical Sciences approved this study (IR.MUBABOL.HRI.REC.1397.108).

### Measurement

#### Social Support Questionnaire (SSQ)

Fleming et al. (1982) developed this questionnaire which comprises 25 items with five subscales (social support from families, neighbors, friends, and general, and view about these supports). The responders would reply to each item as yes/no. The range of the total score was between 0 and 25 [15]. The validated Persian version of SSQ [16] was used in this research. Herein, the *Cronbach’s alpha for the* subscales’ internal consistency ranged from 0.86 to 0.95.

#### Revised Prenatal Distress Questionnaire (NuPDQ)

This questionnaire is a modified version of the PDQ, with both tools being frequently employed for assessing pregnancy stress [17, 18]. NuPDQ consists of 17 items evaluating certain pregnancy-related worries, such as bodily changes, fetal health, physical symptoms, and delivery. The pregnant women rate their level of “worry” on a scale varying from 0 (not at all) to 2 (very much). The total score ranges from 0 to 32. In the current work, *Cronbach’s alpha for* internal consistency of the scale was 0.91. We used the NuPDQ’s validated Persian version with five aspects, namely medical problem, parenting, infant healthy, pregnancy symptom, and fear of childbirth [19].

### Statistical analysis

The descriptive analysis of all the variables (age, parity, gestational age, pregnancy specific-stress, and social support) was presented as percentages, means, and standard deviation. Since the quantitative variables of the study, such as self-care, depression, and fear of COVID-19, followed a normal distribution, we used parametric tests to compare the means. The mean comparison of pregnancy stress and social support between the village residents and city residents was performed utilizing student *t* tests. The correlations between social support and pregnancy-associated stress were measured with Pearson’s test. In order to explore the associations between the five subscales of pregnancy stress, social support was embedded into two linear regression analyses with adjusted and non-adjusted models. Based on each subscale of social support (social support from the side of families, neighbors, friends, as well as the public, along with view about such supports), a number of simple linear regression models were developed. Five variables, including age, place of living, level of education, job, gestational age, and duration of marriage, were considered as covariates in the adjusted models. The Statistical Package for the Social Sciences (SPSS) software (version 18.0 ) was used to analyze the data. A significant level was defined as a *P*-value of *<*0.05.

## Results

Table 1 represents the demographic details in terms of the population and psychological variables based on the place of residence. No significant difference was observed between the pregnant women living in village and those living in city regarding age, education, gestational age, age of marriage, pregnancy stress, and scores of social support. However, the difference was significant in the subscale of belief about the support.

**Table 1:** Demographics and psycho-social variables of the pregnant women attending antenatal care in Babol, Iran, during 2018–2019, by the place of residence

Variables	Living in village No. (%)	Living in city No. (%)	*P*-value*
Age in years ^a^			
18–29	64 (65.3)	58 (67.4)	
30–45	34 (34.7)	28 (32.6)	0.441
Educational level			
Primary and high school	76 (76)	77 (77)	0.052
University	24 (24)	23 (23)	
Parity			
1	36 (36)	44 (44)	0.454
1–2	60 (60)	54 (54)	
≥3	4 (4)	2 (2)	
Job			
Employed	5 (0.5)	3 (0.3)	0.159
Unemployed	95 (95.0)	97 (97)	
Gestational age, M (SD)	24.41 (5.99)	25.49 (6.87)	0.053
Duration of marriage (Years)	6.46 (3.93)	5.33 (4.39)	0.074
**Pregnancy stress**			
Medical and financial problems-related stress	2.05 (1.35)	2.13 (1.20)	0.659
Parenting stress	1.44 (1.40)	1.51 (1.46)	0.730
Infant health stress	1.73 (1.14)	1.88 (1.11)	0.349
Pregnancy symptom stress	2.90 (1.80)	3.14 (1.75)	0.341
Fear of childbirth	2.43 (1.03)	2.17 (1.12)	0.990
Total score of stress (0-25)	11.56 (4.70)	11.65 (4.95)	0.907
**Social Support**			
Friends’ support	2.26 (1.04)	2.26 (0.96)	1.00
Neighbors’ support	1.96 (1.38)	2.00 (1.40)	0.840
Family support	5.85 (1.52)	6.12 (1.24)	0.172
General support	2.69 (1.27)	2.34 (1.27)	0.054
Belief in support	2.72 (1.12)	2.40 (0.96)	0.032*
Total score of support (0-34)	15.48 (3.00)	15.12 (2.91)	0.391

*Chi-square tests to compare the frequencies; t-tests to compare the means; significant level at *P*<0.5

^a^Due to the missing data, the total number of the participants in the age group is less than 100 participants

As shown in Table 2, a negative association existed between the subjects’ level of education and general support (*P*=0.007) as well as neighbor’s support (*P*=0.005). Furthermore, a significantly negative association was seen between the working women and pregnancy stress. There was a significantly negative association between the place of residence and belief in social support (*P*=0.020). On the other hand, a significantly positive association was found between the gestational age of the women and pregnancy stress (*P*=0.025), as well as neighbors’ support (*P*=0.013). However, there was no significant correlation between their age, age of marriage, or the total scores of both social support and pregnancy stress.

**Table 2:** Correlation between the demographics of the pregnant women and the scores of social support and pregnancy stress

Variables	Friends’ support	Neighbors’ support	Family support	General support	Belief support	Total support	Pregnancy stress
**Age** *P*-value	–0.002 0.915	–0.042 0.557	–0.079 0.326	0.029 0.088	0.088 0.215	–0.010 0.390	–0.106 0.137
**Education** *P*-value	–0.30 0.684	–0.121 0.005*	–0.050 0.490	–0.194 0.007*	0.069 0.344	–0.993 0.202	0.044 0.544
**Employed** *P*-value	0.058 0.444	–0.228 0.002*	–0.116 0.111	0.035 0.632	0.113 0.120	–0.061 0.406	–0.258 <0.001*
**Place of living** *P*-value	–0.024 0.736	0.013 0.850	0.105 0.141	–0.111 0.099	–0.164 0.020*	–0.066 0.350	0.035 0.619
**Gestational age** *P*-value	0.056 0.434	0.176 0.013*	0.001 0.994	–0.066 0.355	–0.121 0.088	0.030 0.669	0.155 0.025*
**Age of marriage** *P*-value	–0.974 0.327	0.066 0.383	–0.062 0.413	0.058 0.438	0.031 0.683	0.012 0.874	0.101 0.182

*Significant at *P*<0.5

Table 3 depicts the association between the subscales of social support and pregnancy stress. A significantly positive association was found between neighbors’ social support and the total score of pregnancy stress (*P*<0.001), as well as of the four subscales of medical problems (*P*=<0.001), parenting (*P*=0.025), infant healthy stress (*P*=0.006), and pregnancy symptoms (*P*=0.001). The belief in social support was significantly and negatively correlated with the total pregnancy stress and stress concerning the infant’s health (*P*=0.033) on top of pregnancy symptoms (*P*=0.006). A significantly positive association was observed between the total score of social support and pregnancy stress (*P*=0.011) as well as stress concerning the infant’s health (*P*=0.034). Nevertheless, there was no significant association between the subscales of friends’ and family support and pregnancy stress along with its subscales, including medical problems (*P*<0.001), parenting (*P*=0.025), infant health stress (*P*=0.006), and pregnancy symptoms (*P*=0.001).

**Table 3:** Association between the subscales of social support and pregnancy stress

Social support	Pregnancy stress					
	Medical Problems	Parenting	Infant health	Pregnancy symptoms	Fear of birth	Total stress
**Friends** *P*-value	0.17 0.811	0.029 0.682	0.068 0.342	0.079 0.265	−0.096 0.178	0.919 0.794
Neighborhood *P*-value	0.253 <0.001*	0.159 0.025*	0.193 0.006*	0.240 0.001*	0.120 0.089	0.306 <0.001*
**Family** *P*-value	0.021 0.773	0.152 0.025	0.116 0.101	0.126 0.076	0.006 0.929	0.110 0.122
**General** *P*-value	0.042 0.554	−0.030 0.675	0.129 0.069	0.002 0.971	0.170 0.016	0.072 0.308
**Belief** *P*-value	−0.049 0.492	0.101 0.131	−0.208 0.003*	−0.194 0.006*	−0.003 0.970	−0.151 0.033*
**Total score** **of social support**	0.135 0.056	0.105 0.139	0.150 0.034*	0.131 0.965	0.100 0.158	0.180 0.011*

*Significant at *P*<0.5

Table 4 demonstrates the outcomes of linear regression models on the basis of every single subscale of pregnancy stress as the dependent variables and the total score of social support with its five subscales as independent variables through adjusted regression analyses.

**Table 4:** Associations between pregnancy stress and social support in adjusted models of regressions

Dependent variable (Stress)	Independent variables (Social support)	Adjusted model	
		*B*	Sig
**Medical problem stress**	Friends	0.003	0.974
	Neighbors’ support	0.147	0.047*
	Family support	− 0.304	0.761
	General support	0.067	0.413
	Belief in support	0.043	0.599
	Total score of support	0.098	0.226
**Parenting stress**	Friends’ support	− 0.047	0.602
	Neighbors’ support	0.010	0.902
	Family	0.109	0.162
	General	− 0.008	0.924
	Belief in support	− 0.086	0.282
	Total score of support	0.001	0.966
**Infant healthy stress**	Friends’ support	0.100	0.207
	Neighbors’ support	0.027	0.752
	Family	0.084	0.291
	General support	0.210	0.009*
	Belief in support	− 0.144	0.076
	Total score of support	0.130	0.103
**Pregnancy symptom stress**	Friends’ support	0.113	0.157
	Neighbor’s support	0.203	0.017*
	Family	0.088	0.269
	General	0.066	0.419
	Belief in support	− 0.157	0.054
	Total score of support	0.145	0.071
**Fear of childbirth stress**	Friends’ support	− 0.085	0.289
	Neighbor’s support	0.164	0.046*
	Family	0.028	0.729
	General support	0.221	0.996
	Belief of support	0.058	0.479
	Total score of support	0.172	0.032*
**Total score of** **pregnancy stress**	Friends’ support	0.009*	0.911
	Neighbor’s support	0.172	.004*
	Family	0.066	0.396
	General supper	0.148	0.062
	Belief in support	− 0.055	0.493
	Total score of support	0.155	0.046*

Model adjusted for age, place of living, level of education, job, gestational age, and age of marriage

*Significant at *P*< 0.05

The support of neighbors was the independent variable related to the stress caused by medical problems in the pregnant women regression (*β* = 0.147, *P =* 0.047). The social support of neighbors (*β* = 0.163, *P =* 0.025) as well as family support (*β* = 0.156, *P =* 0.032) were significantly related to parenting stress in the non-adjusted model. The social support of neighbors (*β* = 0.156, *P =* 0.006) and general support were significantly and positively related to the infant’s health. However, the social support belief (*β* = -0.222, *P =* 0.003) was negatively related to the stress in pregnant women. Furthermore, the neighbors’ social support (*β* = 0.306, *P =* 0.001) was positively related to pregnancy stress symptoms in the subjects. The neighbors’ social support (*β* = 0.161, *P =* 0.046) and general support (*β* = 0.221, *P =* 0.006) were positively and significantly related to the fear of childbirth for pregnant women. Finally, in the adjusted model, the neighbors’ social support (*β* = 0.172, *P =* 0.04) and total social support score (*β* = 0.155, *P =* 0.046) were positively and significantly related to the total score of pregnancy stress.

## Discussion

Herein, we found that the total social support was related to pregnancy stress. The findings emphasized that neighbors’ support, as a major social support component, was significantly related to pregnancy stress. The majority of studies have focused on the positive effects of social connections while their negative effects have received little attention in the literature [2]. On the contrary to our findings, a prospective-longitudinal study on 306 women after delivery revealed that perinatal maternal stress was lower in those with a higher total social support [10]. Meanwhile, evidence has confirmed that negative social support can potentially be harmful for health [20].

Now the question is how do social interactions create negative social support? In response, we can say that social relations have both positive and negative aspects. Once there are elements in a person’s social relationships that contribute to solving the person’s problems and reducing tensions, these social relationships are supportive. Negative social support is defined as when a person experiences a lot of tension and discomfort in relationships with others, which not only do not solve their personal problems, but also create interpersonal stress. Negative interactions can include discouraging feelings, making critical comments, invading others’ privacy, interfering with other people’s business, or failing to provide assistance or deliver promises. In the negative social support, either the individual is not able to use the sources of positive social support in interpersonal relationships or the interpersonal elements fail in supporting the individual [10, 21, 22].

In order to answer the question of how social support is positively associated with women’s stress, we assessed the relationship between the subscales of social support and pregnancy stress. It was revealed that only one of these subscales, namely that of neighbors, was significantly and positively correlated with the total score as well as all the subscales of pregnancy stress. The results of regression analysis also confirmed that the neighbors’ support was significantly associated with the total score of social support in pregnancy stress. A few studies have reported the association between neighbors’ support and stress in pregnant women. Inconsistent with our results, a study in USA reported that lower neighborhood quality based on four measures (neighborhood safety/danger, neighborhood disorder, walking environment, overall rating) was related to a higher level of mental stress during pregnancy [23]. Vinikoor-Imler et al. in the USA documented that the neighbors’ conditions of pregnant women were associated with health-related behaviors of these women [24]. Morozumi et al. investigated personal and neighbors’ social support impact on the physical and mental health of 79,210 pregnant Japanese women. The results revealed that only 40% of them agreed to get neighbors’ social support. Additionally, the supportiveness of neighbors was found to be associated with older age, being married, having children, no existing diseases, no obstetric complications, and higher family income. The finding demonstrated the positive neighbors’ social support impact on the mental health of pregnant women [25]. In addition, a study in adults from 60 US communities (*n*=12,716) examined the impact of neighborhood-related stressors and stress-relieving strategies on mental health. This research looked at the link between local stressors, stress coping techniques, and risk of adults’ mental health issues. The study found that a lower likelihood of mental illness in neighboring areas was associated with further stress-buffering mechanisms. People with low levels of social support in neighborhoods were also at a higher risk of elevated levels of social isolation (low average household occupancy) and mental illness [26].

The reason behind the discrepancy between our study results and those of other works, especially regarding the neighbors’ support, is not clear. We could suggest that this be addressed in future research. Nevertheless, it could be associated with certain probable methodological differences between the investigations regarding the difference in the inclusion criteria, different scales, different social cultures, and socioeconomic situation of the participants.

The findings herein can have clinical implications. The current research can suggest researchers to consider all the subgroups of social support, not only the total scores in data analysis, as well as interpretations of social support and stress. Certainly, further research is needed to develop assessment systems that include both merits and downsides of social interactions. In addition, future research should concentrate on developing a theoretical framework for understanding how neighbors’ support influences pregnant stress.

### Strengths and limitations of the study

One of the study’s strengths was that it looked at the association between social support and all its components with pregnancy stress. It also highlighted how one of the subcomponents of social support (neighbor support) was independently and significantly related to pregnancy stress. Meanwhile, a limitation of the study was that it was cross-sectional; therefore, in this work, the association between social support and pregnancy stress did not mean causal. Longitudinal investigations are also required to obtain a better understanding of the effect of social capital, particularly neighbors’ support, on pregnancy stress. Another limitation was the use of self-reporting tools for the participants rather than observation of the interactions with neighbors, which may have caused bias in the results. It could be recommended that further research collect information by directly observing the neighbors’ interactions with pregnant women. The small sample size, low number of employed women, and the use of convenient sampling methods were some other limitations of the study. Ultimately, the women may not represent the general population since our sample was selected from two obstetrics clinics in a referral hospital and a private practice.

## Conclusion

The findings demonstrated that social support does not always result in positive experiences for pregnant women. The study highlighted the negative effects of neighbors’ support on stress in this population. Healthcare providers are recommended to pay further attention to the positive and negative effects of social support during the pregnancy period. More research, such as interventional planning, would be conducive to reducing the effect of negative interactions of pregnant women during prenatal care.

## Data Availability

Datasets used in the current study are available from the corresponding author on reasonable request.
